# Inferring Single-Cell 3D Chromosomal Structures Based on the Lennard-Jones Potential

**DOI:** 10.3390/ijms22115914

**Published:** 2021-05-31

**Authors:** Mengsheng Zha, Nan Wang, Chaoyang Zhang, Zheng Wang

**Affiliations:** 1 School of Computing Sciences and Computer Engineering, University of Southern Mississippi, 118 College Dr, Hattiesburg, MS 39406, USA; mengsheng.zha@usm.edu (M.Z.); chaoyang.zhang@usm.edu (C.Z.); 2 Department of Computer Science, New Jersey City University, 2039 Kennedy Blvd, Jersey City, NJ 07305, USA; nwang1@njcu.edu; 3 Department of Computer Science, University of Miami, 1364 Memorial Drive, Coral Gables, FL 33124, USA

**Keywords:** 3D genome, single-cell Hi-C, 3D chromosomal structure, Lennard-Jones potential

## Abstract

Reconstructing three-dimensional (3D) chromosomal structures based on single-cell Hi-C data is a challenging scientific problem due to the extreme sparseness of the single-cell Hi-C data. In this research, we used the Lennard-Jones potential to reconstruct both 500 kb and high-resolution 50 kb chromosomal structures based on single-cell Hi-C data. A chromosome was represented by a string of 500 kb or 50 kb DNA beads and put into a 3D cubic lattice for simulations. A 2D Gaussian function was used to impute the sparse single-cell Hi-C contact matrices. We designed a novel loss function based on the Lennard-Jones potential, in which the ε value, i.e., the well depth, was used to indicate how stable the binding of every pair of beads is. For the bead pairs that have single-cell Hi-C contacts and their neighboring bead pairs, the loss function assigns them stronger binding stability. The Metropolis–Hastings algorithm was used to try different locations for the DNA beads, and simulated annealing was used to optimize the loss function. We proved the correctness and validness of the reconstructed 3D structures by evaluating the models according to multiple criteria and comparing the models with 3D-FISH data.

## 1. Introduction

Compared to the study of the genome from a one-dimensional (1D) or sequence perspective, the research of spatial folding of the 3D structure of the DNA has been increasingly recognized as important [1,2,3] because the spatial locations of genes, enhancers, promoters, and lncRNAs are essential for biological functions such as transcription, replication, DNA repair, and chromosome translocation [4,5]. Based on the spatial folding of the DNA, researchers have defined on average 1 Mb long topologically associating domains (TADs) on the genome [6], which can be the structural and functional units of the genome. Research such as the reconstruction of high-resolution 3D genome structures [7] and family classification of TADs [8] lead to a deeper understanding of how the genome functions.

Empowered by the methods of chromosome conformation capture including 3C, 4C, 5C, and Hi-C, the technology of genome conformation detection is applicable to not only a selection of loci of chromosomes but also higher-order structures such as the entire genome. Specifically, 3C methods can detect the proximate or in-contact relationship in the three-dimensional (3D) space between a single location in the chromosome and other selected genomic regions (single vs. selected) [9,10,11,12,13,14,15,16,17,18]; 4C methods can detect the same in-contact relationship between one genomic region and all other genomic regions (one vs. all) [19,20,21,22,23,24,25]; 5C can detect the spatial proximities between selective genomic regions and other selective genomic regions (many vs many) [26,27,28,29,30,31]; and Hi-C, as one of the latest chromosome conformation capturing methods, can detect genome-wide all-to-all spatial in-contact relationships [32,33,34,35,36,37,38,39,40,41,42,43,44,45,46,47,48,49,50,51,52,53,54].

Each cell contains a complete genome, which consists of multiple chromosomes. Each chromosome is a two-strand (+ and −) string of nucleotides (A, G, C, and T). The Hi-C experiment can detect which two locations in the same chromosome (intra-chromosomal) or two different chromosomes (inter-chromosomal) are spatially proximate or in contact in the 3D space. Usually, the entire chromosome is represented using a beads-on-a-string approach, in which a certain number of nucleotides, for example, 500 kb (kilo DNA base pairs), is considered a bead, and the chromosome is considered a long string of 500 kb DNA beads. The 500 kb length for each bead is also called the resolution of the 3D chromosome structure. The lower the length of each bead is, the higher resolution the reconstructed 3D structure is at, and a higher resolution leads to a higher number of beads for the same chromosome.

Using the Hi-C method as an example, the intra- and inter-chromosomal contact maps can be generated to indicate the number of Hi-C contacts detected between two regions of the same or different chromosome(s). Based on a Hi-C contact map, the 3D structures of the DNA can be reconstructed. The 3D chromosomal structures of yeast were reconstructed to find relationships between the function and structure of the eukaryotic genome [55]. The computational module named Integrative Modeling Platform (IMP) can be used to measure and evaluate the 3D structure of genomic regions and the whole genome. Specifically, [56] used IMP to model 3D chromosomal structures, from which the authors found the characteristics of each chromatin and their relationships to gene expression. The interaction frequencies (IF) detected by 5C and Hi-C have both been used to computationally model and analyze three-dimensional chromatin organization in [57]. Data noise and structural changeability were used to evaluate the mean-field restraint-based methods of 3D genome structure reconstruction and were used to verify the limitations of the 3D modeling methods [58]. The semi-definite programming techniques have been applied in ChromSDE, a method to figure out the best structure that matches the observed data and discover the optimal parameters to transform Hi-C contact numbers to Euclidean distance in the 3D space using golden section search [59]. BACH, a Bayesian 3D genome structure reconstructing algorithm based on Hi-C data, together with its updated version BACH-MIX have been used to search the structural variations of chromatin in a cell population and find the consensus 3D chromosomal structure. Based on the experiments of applying BACH and BACH-MIX to mouse embryonic stem cells, BACH and BACH-MIX were considered to have the potential to accurately reconstruct the chromosomal structures of mammalian cells [60]. [61] claimed that Poisson models can better reconstruct chromosomal structures in comparison to multidimensional scaling (MDS)-based methods since Poisson models produce more reproducible structures than MDS-based methods. Last but not the least, the 3D chromosomal reconstruction method named MOGEN uses a data-driven scoring function to model the in-contact and not-in-contact relationships between pairs of DNA segments in the same or different chromosome(s) [62]. miniMDS [63] builds 10 kb resolution chromosomal structures by partitioning a Hi-C dataset, performing high-resolution MDS on each partition, and reassembling the inferred structure for each partition using low-resolution MDS. 3D-GNOME 2.0 [64] can predict the alternations of chromosomal 3D structures driven by structural variants given the input of Hi-C data, ChIA-PET data, CTCF interactions, or CTCF and RNPII interactions. GEM [65] reconstructs 3D chromosomal structures by integrating Hi-C data with biophysical feasibility and directly embeds the neighboring affinities from the Hi-C space into the 3D Euclidean space. GEM-FISH [66] uses both Hi-C and FISH data and a polymer biophysical model to infer 3D chromosomal structures and reveals spatial distribution patterns of super-enhancers. ChromStruct4 [67] is a multiresolution method that uses modified-bead-chain representation of chromatin and Monte Carlo sampling to build a set of 3D configurations that fit the Hi-C data and prior knowledge. PGS [68] can deconvolve the population-cell Hi-C data and generate a population of distinct diploid 3D genome structures that match inter-chromosomal contact patterns.

The Hi-C method mentioned above is a biochemistry experiment conducted on a population of cells, usually in the range of 10^5^ cells. Therefore, it is often called population-cell Hi-C or bulk Hi-C. Because each cell has a complete genome, the population Hi-C contacts are a consensus dataset over a pool of cells. In other words, millions of Hi-C contacts can usually be generated. Therefore, the common idea of the population-Hi-C-based methods for reconstructing 3D chromosome structure is that a higher number of contacts detected between a pair of DNA beads indicates a shorter Euclidean distance between the two beads in the reconstructed 3D structure.

The recent development of biochemistry technology began to focus on individual cells or single cells because that allows scientists to understand the accurate knowledge about every single cell instead of a consensus result from a population of cells. The same trend has also happened in the field of chromosome conformation capturing. Recently, single-cell Hi-C techniques have been invented and applied to mouse and human cells [69,70,71,72,73,74]. The bioinformatics community would naturally want to design algorithms to model the 3D structure of a single cell because of the potential significance and novelty of the research. However, modeling 3D chromosomal structures based on single-cell Hi-C data is a difficult computational task because the number of Hi-C contacts observed from a single-cell Hi-C experiment is usually much less than the ones generated from a population-cell Hi-C experiment. In other words, zero is the dominant value in the single-cell Hi-C contact matrices, making the matrices highly sparse. [75] used a modified Bayesian inferential structure determination (ISD) framework to address this challenge. [69] modeled the 3D chromosomal structures based on single-cell Hi-C data using molecular dynamics and simulated annealing. SIMBA3D [76] is a Bayesian multiscale approach for inferring single-cell chromosomal structures using population-cell Hi-C data as prior. None of these methods considered the influence that a single-cell Hi-C contact can generate on the sequentially neighboring bead pairs. SCL [77] uses a 2D Gaussian function to impute the influence that a single-cell Hi-C contact generates to its sequentially neighboring bead pairs. In this research, we adapted the same 2D Gaussian imputation approach but used the Lennard-Jones potential to model the single-cell 3D chromosomal structures, which to the best of our knowledge is the first attempt in the field.

Polymer physics has been applied to model and analyze chromatin structures. In [78], the authors modeled the structure of TADs based on loop extrusion and found that the cis-acting loop-extruding factors, likely cohesins, form large loops but stall at the TAD boundaries. Also based on loop extrusion and using only information about the binding locations of CTCF, the authors of [79] accurately predicted how the genome will fold. The “strings and binder switch” (SBS) model can model the chromatin folding changes based on the binding site distribution, binder concentration, or binding affinity in a switch-like fashion, which has been used in [80] to capture the complexity of chromatin folding. The authors of [81] at first used machine learning to predict binding locations based on population-cell Hi-C data and then also used the phrase-separation SBS model to indicate chromatin folding variability across single cells. Other studies that also used phrase-separation models to build and analyze 3D DNA structures include [80,82,83].

The Lennard-Jones potential has been widely used for molecular dynamics simulations [84,85,86,87,88] as its mathematical formula can very closely mimic the real repulsive and attractive forces between molecules. [89] used the Lennard-Jones potential to model the 3D structures of proteins. In [82], every 10 kb of DNA is represented as a monomer, and a truncated Lennard-Jones-like potential is used to model the energy between monomers. The authors of [79] used the Lennard-Jones potential to model both the intermonomer attractions and external crushing forces during condensation when they used loop extrusion to model the 3D structures of wild-type and engineered genomes. However, the Lennard-Jones potential has not been used to model genome 3D structures based on extremely sparse single-cell Hi-C data.

## 2. Results and Evaluations

### 2.1. Inferred 3D Structures of the X-Chromosome of a Mouse TH1 Cell

We downloaded the single-cell Hi-C data of individual male mouse TH1 cells from [69], in which high-quality single-cell Hi-C data were generated on 10 cells. The single-cell Hi-C experiment on cell 1 has the highest quality based on [69]. Therefore, it was used in this research. There are 616 single-cell intra-X-chromosome Hi-C contacts in the dataset. After removing self-contacts, 438 single-cell Hi-C contacts were kept and used as the input of our algorithm.

**Figure 1:**
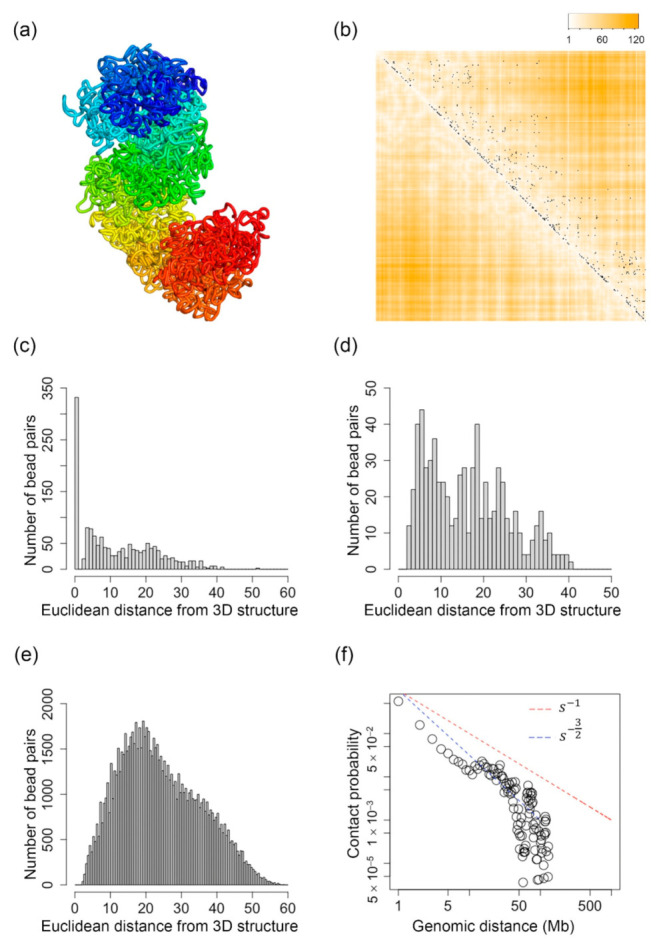
(**a**) The inferred 3D structure of the X-chromosome of a mouse Th1 cell at 50 kb resolution when n in Equation (3) equals 2. (**b**) Each black dot indicates one single-cell Hi-C contact. The darker orange color indicates higher Euclidean distances parsed from the inferred 3D structure, and the white color indicates small Euclidean distances parsed from the inferred 3D structure. (**c**) The distribution of the number of bead pairs over different values of Euclidean distances parsed from the inferred structure when $\theta_{i,j}=1$. (**d**) The distribution of the number of bead pairs over different values of Euclidean distances parsed from the inferred structure when $\theta_{i,j}\in \left(0.7,\;1\right)$. (**e**) The distribution of the number of bead pairs over different values of Euclidean distances parsed from the inferred structure when $\theta_{i,j}\in \left(0,\;0.7\right]$. (**f**) The contact probability of bead pairs over different values of genomic distances.

Figure 1a shows the 3D structure of the X-chromosome at 50 kb resolution inferred by our algorithm. In Figure 1b, each black dot represents a single-cell Hi-C contact detected for the Th1 X-chromosome. The heatmap overlapped with the single-cell Hi-C contacts represents the Euclidean distance of every bead pair that is parsed from the inferred 3D structure. Lighter orange color represents shorter distances, whereas darker orange color represents longer distances. Therefore, if we see that white or lighter colors are mostly overlapped with black dots (single-cell Hi-C contacts), that means the reconstructed 3D structure makes sense or fits the single-cell Hi-C data. From Figure 1b, we can see that all of the reconstructed 3D structures contain large regions that well fit the single-cell Hi-C data.

Figure 1c–e show the number of bead pairs that have different ranges of Euclidean distances based on the reconstructed 3D structures when the $\theta_{i,j}$ value is 1, (0.7, 1) and (0, 0.7], respectively. We performed a Mann–Whitney U test, and the p-value for the distributions in Figure 1c,d is 4.1 × 10^−24^, the p-value for the distributions in Figure 1c,e is 5.2 × 10^−298^, and the p-value for the distributions in Figure 1d,e is 7.2 × 10^−69^. These small p-values indicate that the distributions are statistically different. We can see that for all the structures shown in Figure 1, the bead pairs that have $\theta_{i,j}=1$ usually have much shorter Euclidean distances compared to the bead pairs that have $\theta_{i,j}$ values in the ranges of (0.7, 1) and (0, 0.7]. The value of $\theta_{i,j}$ is directly related to single-cell Hi-C contacts, that is, when there is a single-cell Hi-C contact existing for a bead pair, its $\theta_{i,j}=1$, and a small $\theta_{i,j}$ value indicates that the bead pair is far away from the other bead pairs that do have single-cell Hi-C contacts. Therefore, the findings in Figure 1c–e indicate that our reconstructed 3D structures well fit the single-cell Hi-C contact data.

Figure 1f shows the relationship between genomic distance, which is represented as s, and the contact probability on a logarithmic scale. For comparison purposes, we plotted two straight lines indicating $s^{-1}$ and $s^{-\frac{3}{2}},\;$ which are the hallmarks of a fractal globule structure and an ideal equilibrium globule structure, respectively [75]. The fractal globule packing of chromosomes was proposed in [90] and [91] based on population-based Hi-C data. Figure 1f shows that the reconstructed 3D structure is more similar to an equilibrium globule structure with genomic distance <10 Mb and in between fractal and equilibrium globule structures when genomic distance is larger than 20 Mb and smaller than 40 Mb. Our results indicate that the single-cell chromosomal structures have a more complicated or mixed packing of both fractal and equilibrium globules, which also fits the finding from another research [75].

To test the influence of the well depth in the Lennard-Jones potential on the reconstructed 3D chromosomal structures, we benchmarked four different values of n in Equation (3). In our newly-designed loss function, the depth of the energy well of the Lennard-Jones potential is related to $\theta_{i,j}{}^{n}$, and the value of $\theta_{i,j}$ is between zero and one, i.e., $0<\theta_{i,j}\leq 1$, indicating either the existence of a single-cell Hi-C contact between bead *i* and *j* or an imputed value based on a 2D Gaussian function. Details can be found in Materials and Methods. Therefore, the value of n, which is an integer, will generate different outcomes when $\theta_{i,j}=1$ or $\theta_{i,j}<1$. For example, when $\theta_{i,j}=1$, no matter what value we assign to n, $\theta_{i,j}{}^{n}$ will always be 1. However, when $\theta_{i,j}=0.1$, a larger n will make $\theta_{i,j}{}^{n}$ much smaller.

**Figure 2:**
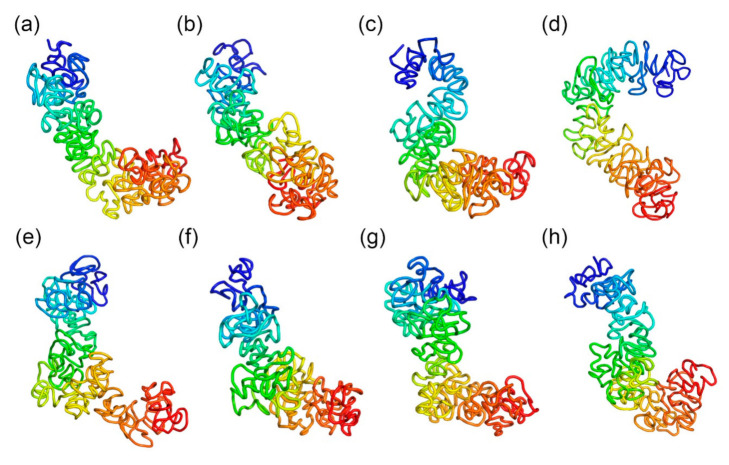
(**a**–**d**) are the 3D structures of the X-chromosome of a TH1 cell when r_m_ in Equation (3) equals 8.976 and n in Equation (3) equals 1, 2, 3, and 4, respectively. (**e**–**h**) are the 3D structures of the X-chromosome of a TH1 cell when r_m_ in Equation (3) equals 8 and n in Equation (3) equals 1, 2, 3, and 4, respectively.

Figure 2 shows the inferred 3D structures for the TH1 X-chromosome with *n* = 1, 2, 3, and 4. We have also tested two different values of $r_{m}$ in Equation (3). Particularly, Figure 2a–d shows four inferred structures with *n* = 1, 2, 3, and 4 and r_m_ = 8.976, and Figure 2e–h are four inferred structures with *n* = 1, 2, 3, and 4 and r_m_ = 8. It can be found that the overall topologies of the inferred structures are stable, particularly when *n* = 1, 2, and 3. We calculated the Pearson’s correlation between $\theta_{i,j}$ values and pairwise Euclidean distances parsed from these inferred 3D structures, which will be discussed in detail in the next section.

### 2.2. Pearson’s Correlation Between $\theta_{i,j}$ and Euclidean Distances Parsed from the Inferred 3D Structures

We tested different values of *n* in Equation (3) including 1, 2, 3, and 4 on the same mouse TH1 cell, and then calculated the Pearson’s correlation coefficients between the $\theta_{i,j}$ values and the Euclidean distances parsed from the inferred 3D structures. A higher $\theta_{i,j}$ value, that is, closer to 1, indicating a single-cell Hi-C contact between the bead pairs or being adjacent to other bead pairs that have single-cell Hi-C contacts, should correspond to a smaller Euclidean distance between the bead pairs in the reconstructed 3D structure. Therefore, a negative correlation is expected, and the smaller the correlation value is, the better the reconstructed 3D structures fit the $\theta_{i,j}$ values.

The Pearson’s correlations for the 3D structures shown in Figure 2a–d are −0.15, −0.21, −0.15, and −0.02, respectively. The *p*-values of these correlation values are 0.0 except for the last one (−0.02) being 10^−7^. The Pearson’s correlations for the 3D structures shown in Figure 2e–h are −0.02, −0.26, −0.14, and −0.16, respectively. The *p*-values of these correlation values are 0.0 except for the first one (−0.02) being 10^−7^. These Pearson’s correlation values were calculated on the structures at 500 kb resolution, and each of the 3D structures consists of 333 beads. Therefore, the Pearson’s correlations were calculated on 55278 bead pairs since we used half of the $\theta_{i,j}$ and distance matrices and excluded the beads on the diagonal lines. This makes all of the correlation values statistically significant, although the absolute values of the correlations are not very high. Since the largest correlation value is achieved when *n* = 2, we used *n* = 2 for all of the other structures, and 2 is used as the default value for *n* in our software.

### 2.3. Comparison with Existing Tools

**Figure 3:**
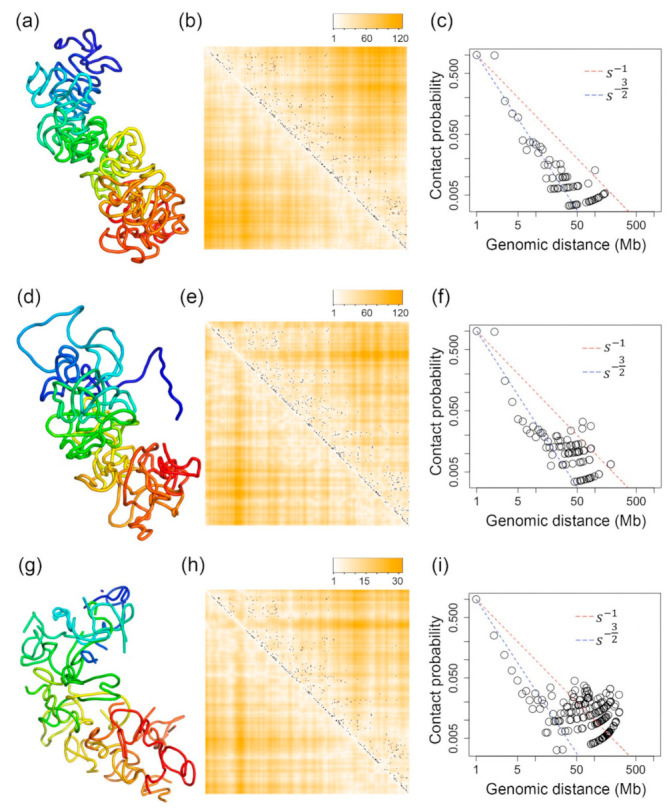
(**a**–**c**) The 3D structure of the X-chromosome of a TH1 cell that is inferred by our Lennard-Jones method and its evaluations. (**d**–**f**) The 3D structure of the X-chromosome of a TH1 cell that is inferred by SCL and its evaluations. (**g**–**i**) The 3D structure of the X-chromosome of a TH1 cell inferred by nuc_dynamics and its evaluations. (**a**,**d**,**g**) are the 3D structures inferred by our Lennard-Jones method, SCL, and nuc_dynamics. (**b**,**e**,**h**) Each black dot indicates one single-cell Hi-C contact. The darker orange color indicates higher Euclidean distances parsed from the inferred 3D structure, and the white color indicates small Euclidean distances parsed from the inferred 3D structure. (**c**,**f**,**i**) The contact probability of bead pairs over different values of genomic distances.

We compared the 3D structures inferred by our method with the structures inferred by SCL [77] and nuc_dynamics [74]. Figure 3a shows the 3D structure inferred from the method presented in this paper. Figure 3d,g are the 3D structures of the same chromosome but inferred by SCL and nuc_dynamics, respectively. All of these structures were inferred based on the same single-cell Hi-C data of the X-chromosome of a mouse TH1 cell as used in Figure 1 and Figure 2. For Figure 3c,f, two beads being in contact is defined as having a Euclidean distance <8, and for Figure 3i, it is a Euclidean distance <0.5 since the structures inferred by different tools are in different scales.

**Figure 4:**
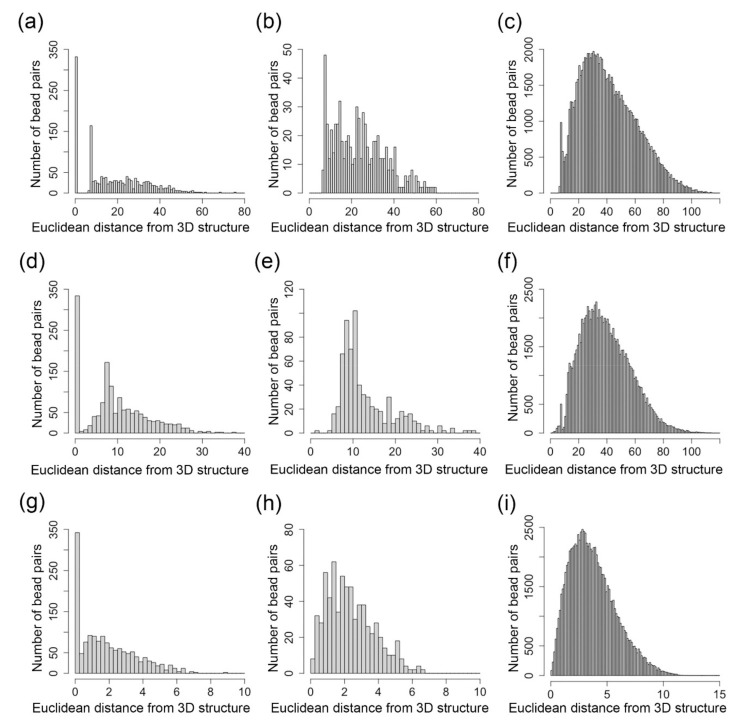
(**a**–**c**) Distributions of the number of nodes for the 3D structure inferred by our Lennard-Jones method. (**d**–**f**) Distributions of the number of nodes for the 3D structure inferred by SCL. (**g**–**i**) Distributions of the number of nodes for the 3D structure inferred by nuc_dynamics. (**a**,**d**,**g**) The distribution of the number of bead pairs over different values of Euclidean distances parsed from the inferred structure when $\theta_{i,j}=1$. (**b**,**e**,**h**) The distribution of the number of bead pairs over different values of Euclidean distances parsed from the inferred structure when $\theta_{i,j}\in \left(0.7,\;1\right)$. (**c**,**f**,**i**) The distribution of the number of bead pairs over different values of Euclidean distances parsed from the inferred structure when $\theta_{i,j}\in \left(0,\;0.7\right]$.

Figure 4 shows the number of bead pairs that have different ranges of Euclidean distances based on the reconstructed 3D structures when $\theta_{i,j}$ is 1, (0.7, 1) and (0, 0.7). We performed a Mann–Whitney U test, and the *p*-value for the distributions in Figure 4a,b is 9.5 × 10^−22^, the *p*-value for the distributions in Figure 4a,c is 0, and the *p*-value for the distributions in Figure 4b,c is 3.4 × 10^−122^. The *p*-value for the distributions in Figure 4d,e is 1.24 × 10^−28^, the *p*-value for the distributions in Figure 4d,f is 0, and the *p*-value for the distributions in Figure 4e,f is 0. The *p*-value for the distributions in Figure 4g,h is 9.8 × 10^−18^, the *p*-value for the distributions in Figure 4g,i is 1.49 × 10^−266^, and the *p*-value for the distributions in Figure 4h,i is 5.85 × 10^−75^. These small *p*-values indicate that these tools can model the bead pairs with different range of $\theta_{i,j}$ in a statistically different way.

Two large regions in the X-chromosome have no single-cell Hi-C data. One of these two regions exists at the beginning of the X-chromosome, which is corresponding to beads 1–11 (the dark blue region in the inferred 500 kb resolution 3D structures), whereas the other region is corresponding to beads 48–66 (the light blue region in the inferred 3D structures). Single-cell Hi-C experiments cannot work at these telomere or centromere regions. Therefore, the single-cell Hi-C data related to these regions are missing. Although SCL also uses the same 2D Gaussian function to impute for missing Hi-C values, the 3D structures for these two regions inferred by SCL are not folded—see Figure 3d. For nuc_dynamics, the software directly removes the 3D structures for this type of region. This is why the 3D structure for the X-chromosome is not continuous in Figure 3g. However, our Lennard-Jones-based method can smoothly fold the structures for these regions—see Figure 3a.

### 2.4. Inferred 3D Structures of the Active and Inactive X-Chromosomes of a Human GM12878 Cell

**Figure 5:**
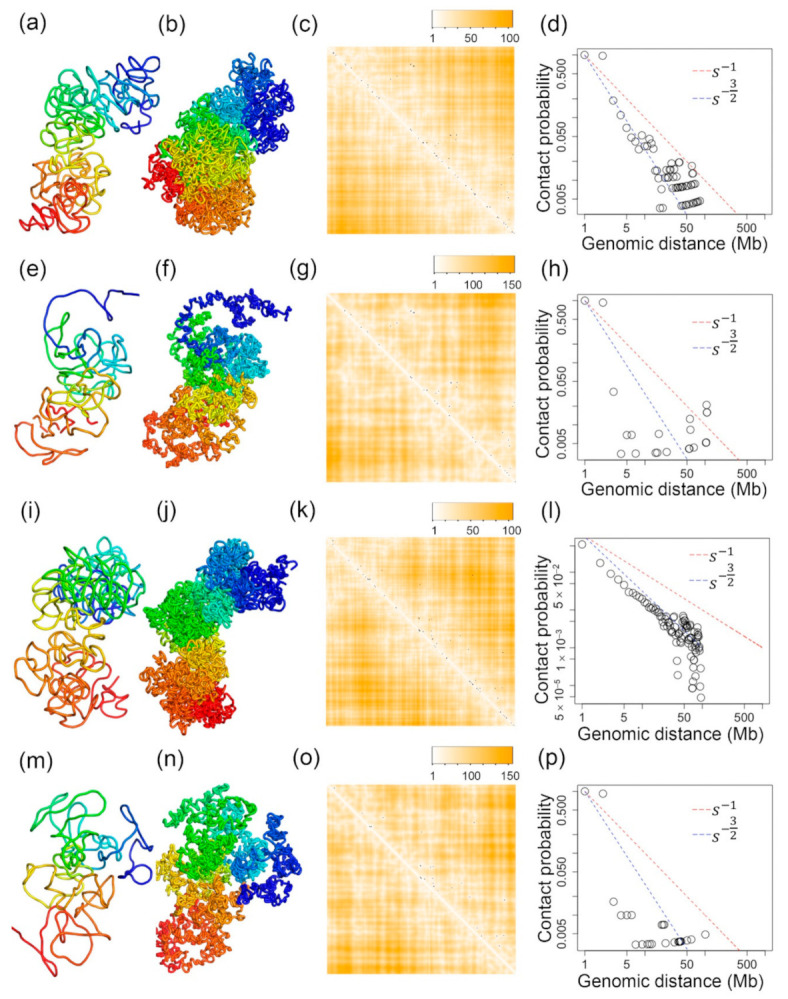
(**a**,**i**) The 500 kb resolution 3D structures of the inactive and active X-chromosome of a GM12878 cell, which is inferred by our Lennard-Jones method, respectively. (**e**,**m**) The 500 kb resolution 3D structures of the inactive and active X-chromosome of a GM12878 cell, which is inferred by SCL, respectively. (**b**,**f**,**j**,**n**) are the 50 kb resolution structures corresponding to (**a**,**e**,**i**,**m**). (**c**,**d**) are the evaluations for (**a**). (**g**,**h**) are the evaluations for (**e**). (**k**,**l**) are the evaluations for (**i**). (**o**) and (**p**) are the evaluations for (**m**).

We reconstructed and evaluated the 3D structures of the two X-chromosomes of a human GM12878 cell. The single-cell Hi-C data were downloaded from [73] with file name GSM3271349_gm12878_03.clean.con.txt.gz. The paper also released imputed single-cell Hi-C data. However, to test the ability of the tools to handle extremely sparse single-cell Hi-C data, we used the original Hi-C data—see Figure 5c for example.

The structures in Figure 5a,e are for the inactive X-chromosome, inferred by our Lennard-Jones method and SCL, respectively. The structures in Figure 5i,m are for the active X-chromosome, inferred by our Lennard-Jones method and SCL, respectively. Figure 5b,f,j,n show the 50 kb resolution structures corresponding to Figure 5a,e,i,m. The inactive X-chromosome is usually silenced in terms of gene expression. The transcripts of lncRNA Xist spread onto one of the two X-chromosomes of female human cells and then alter the 3D structure of the X-chromosome into a highly compacted structure so that the transcriptions on that X-chromosome are stopped [92]. To verify whether the reconstructed 3D structures fit the biological meaning, we calculated the radius of gyration of the 3D structures of inactive and active X-chromosomes. The radius of gyrations of inactive and active structures inferred by our Lennard-Jones method, i.e., Figure 5a,i, are 0.42 and 0.45, respectively. The radius of gyration is a measure of the compactness of a 3D structure. The smaller radius of gyration for the 3D structure of inactive X-chromosome indicates higher compactness of that structure, which fits the biological meaning of inactive X-chromosome and further proves the correctness of our reconstructed 3D structures.

From Figure 5d,h,l,p, it can be found that for extremely sparse single-cell Hi-C data, the 3D structures generated by our Lennard-Jones method still maintain fractal or equilibrium globule structures—see Figure 5d,l—whereas the structures generated by SCL have deviated from fractal or equilibrium globule structures— see Figure 5h,p. This indicates the advantage of our Lennard-Jones method for generating stable structures based on extremely sparse single-cell Hi-C data.

### 2.5. Inferred 3D Structures of the Chromosome 3 of a Mouse Oocyte Cell

**Figure 6:**
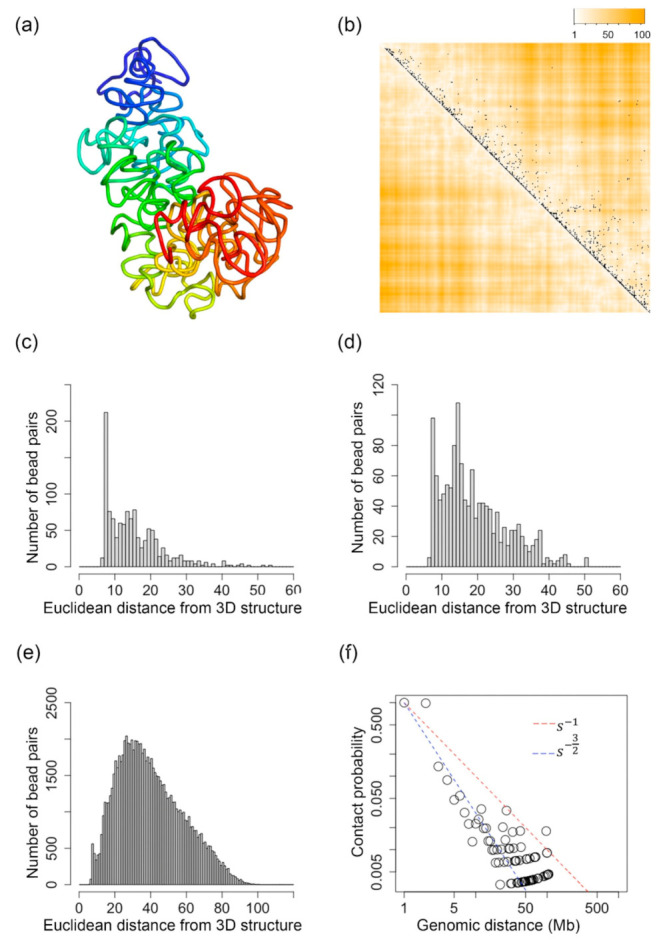
(**a**) The inferred 3D structure of the chromosome 3 of a mouse oocyte cell at 500 kb resolution. (**b**) Each black dot indicates one single-cell Hi-C contact. The darker orange color indicates higher Euclidean distances parsed from the inferred 3D structure, and the white color indicates small Euclidean distances parsed from the inferred 3D structure. (**c**) The distribution of the number of bead pairs over different values of Euclidean distances parsed from the inferred structure when $\theta_{i,j}=1$. (**d**) The distribution of the number of bead pairs over different values of Euclidean distances parsed from the inferred structure when $\theta_{i,j}\in \left(0.7,\;1\right)$. (**e**) The distribution of the number of bead pairs over different values of Euclidean distances parsed from the inferred structure when $\theta_{i,j}\in \left(0,\;0.7\right]$. (**f**) The contact probability of bead pairs over different values of genomic distances.

Figure 6 shows the inferred 3D structure and its evaluations for the chromosome 3 of a mouse oocyte cell. The single-cell Hi-C data was downloaded from [52] (144_oocyte_SN). We performed a Mann–Whitney U test for the distributions of the number of bead pairs with different $\theta_{i,j}$ values, and the p-value for the distributions in Figure 6c,d is 2.6 × 10^−27^, the p-value for the distributions in Figure 6c,e is 0, and the p-value for the distributions in Figure 6d,e is 0.

### 2.6. Validation with 3D-FISH

3D Fluorescence in situ hybridization (FISH) is a type of biochemistry experiment that can detect the distance between pairs of genomic regions. Therefore, by comparing our inferred 3D chromosomal structures with the results of 3D-FISH, we can evaluate the accuracy of the inferred 3D genome structures. We calculated the Pearson’s correlation between the distances of eight probe pairs parsed from the inferred 3D structures and the distances between the same pairs of eight probes detected by 3D-FISH [93]. These eight probe pairs are located on chromosomes 3 and 11. The evaluation was conducted on the reconstructed 3D structure of the TH1 cell at 500 kb resolution. Higher Pearson’s correlation values here indicate that the inferred 3D structure better fits the results from 3D-FISH. We found that when *n* = 1 and r_m_ = 8.976 the 3D structure inferred by our Lennard-Jones method has a Pearson’s correlation of 0.22 to the 3D FISH data.

## 3. Discussion

This research explores the possibility of using the Lennard-Jones potential to reconstruct the 3D structures of chromosomes based on single-cell Hi-C data. Compared to population-cell Hi-C data, single-cell Hi-C contact data are very sparse. This makes it a challenging computational problem because traditional algorithms usually need to convert the number of Hi-C contacts into a target distance, and zero contact would be converted to infinity.

Some studies use Lennard-Jones to model the 3D structures of proteins, e.g., [89]. In those studies, the Lennard-Jones potential was used to model the attractive and repulsive forces between different types of protein residues. However, when we model the 3D structure of chromosomes, each bead is considered to represent *m* DNA base pairs, where *m* is the resolution that can be 500 kb or 50 kb in this research. These DNA bead pairs may not have the clearly defined attractive or repulsive forces as residues in a protein have. Therefore, it may not be possible to infer chromosomal 3D structures by directly modeling the attractive or repulsive forces between bead pairs using the Lennard-Jones potential.

Therefore, we used the well depth of the Lennard-Jones potential to model the confidence for a bead pair to maintain an in-contact distance or the stability of the in-contact relationship. For the bead pairs that we observe a single-cell Hi-C contact for, our loss function assigns stronger confidences or deeper well depths in the Lennard-Jones potential. This makes these bead pairs have a strong potential to maintain such a short distance during the Monte Carlo simulations. For the bead pairs that are far away from the bead pairs that do have single-cell Hi-C contacts, our loss function assigns lower confidences by assigning shallower well depths in the Lennard-Jones potential. The degree of being smaller can be controlled by the value of *n* in Equation (3): a larger *n* will make the well depth increasingly smaller. In other words, for the bead pairs that have small $\theta_{i,j}$ values, we do not force a long distance between the beads, but make their in-contact distances harder to be maintained or easier to be broken in the Monte Carlo simulations.

The results shown in this study demonstrated that this strategy worked. We tested different values of *n* influencing the well depth in the Lennard-Jones potential and conducted evaluations from multiple perspectives. We also cross-validated the inferred 3D structures with the data obtained by 3D-FISH, another biochemistry experiment that can detect 3D proximities of multiple DNA segments. We applied our methods to active and inactive X-chromosomes in a female human cell. The reconstructed 3D structure for the inactivate X-chromosome has a smaller radius of gyration, which fits the biological meaning of X-chromosome inactivation. We also found that our method can keep the inferred 3D structure a mix of fractal and equilibrium globule structure even for extremely sparse single-cell Hi-C data.

## 4. Materials and Methods

### 4.1. Introduction to Negative Potential Energy

**Figure 7:**
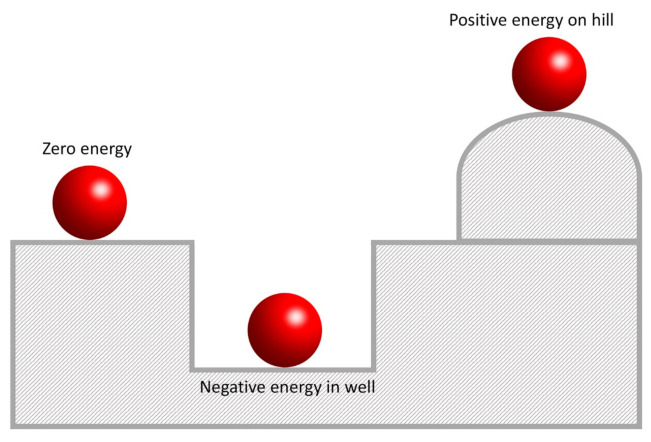
A diagram showing the negative, positive, and zero energy.

The negative potential energy is explained by the three red balls in three different gravitational potential energy statuses in Figure 7. In a flat horizontal plane, the red ball will be stable, that is, it will not move unless pushed by a source of external force that provides some kinetic energy. This situation is called zero energy status. If a ball is located on the top of the hill, gravity will make the ball start rolling down at an increasing speed. During this process, the red ball would emit potential energy so that this situation is labeled as a positive energy status. In the well, the red ball needs to absorb some kinetic energy to start moving and eventually jumps out of the well when it gets enough kinetic energy.

**Figure 8:**
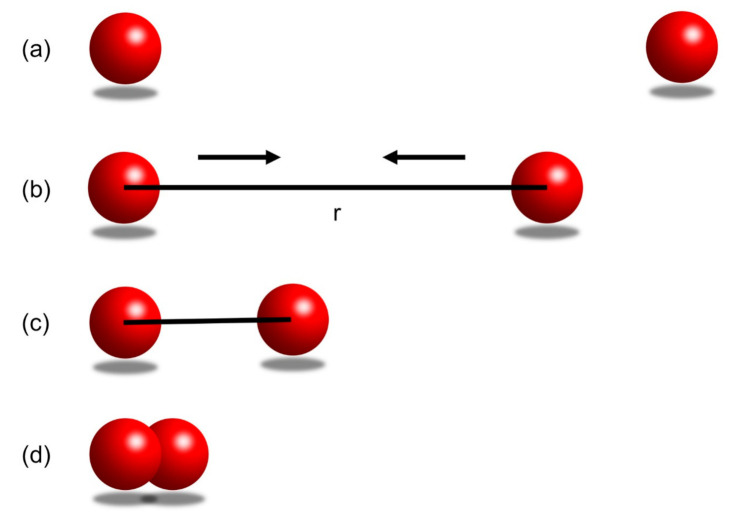
(**a**) The balls are not interacting as they have an infinite distance. (**b**) Two balls are brought closer with minimum energy to a distance of r. The two balls have an attractive force between them. (**c**) The two balls are brought closer by the attractive force between them until they reach an equilibrium distance, at which they reach the sminimum bonding potential. (**d**) External energy pushes the two balls even closer. A repulsive force tries to push the two balls apart, and the repulsive force is greater than the attractive force.

In Figure 8a, the two balls do not interact when there is a long distance between them. In Figure 8b, when the two balls are bought closer to a distance r, they will start to attract each other, i.e., there is an attractive force between them. In Figure 8c, at an even closer distance, the two balls will first accelerate towards each other due to the dominant attractive force, and then reach an equilibrium distance at which the minimum bonding potential is reached. In Figure 8d, when the two balls are getting further closer to each other (for example, pushed by an external source of energy), i.e., the distance between them is less than the equilibrium distance described in (c), the repulsive force will dominate and keeps increasing to try to push the two balls away.

### 4.2. The Lennard-Jones Potential

**Figure 9:**
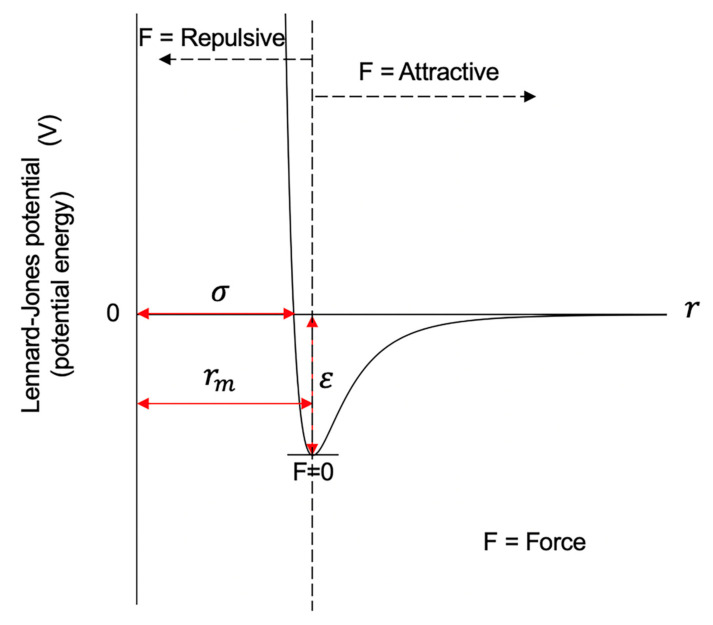
A diagram showing the curve of the Lennard-Jones potential.

The Lennard-Jones potential is a mathematical formula that can be used to model the potential energy and interactions between two atoms or molecules. Figure 9 shows the curve of the Lennard-Jones potential and the repulsive and attractive forces. The *x*-axis in the plot indicates the distance between two particles, and the *y*-axis indicates the inter-particle potential or potential energy. As shown in Figure 9, when the distance between two particles is at the equilibrium distance or r_m_, the force is zero, and the potential energy reaches its minimum, that is, it is at the bottom of the energy well. When the distance increases from the equilibrium distance, the force between the two particles will be the attractive force. Meanwhile, the potential energy increases and gets closer to zero. When the distance decreases from the equilibrium distance, the repulsive force will be gradually stronger.

The depth of the well indicates how strongly the two particles attract each other. As shown in Figure 9, the deeper the well is, the more external energy the ball would need to jump out of the well, that is, the harder it is to get the ball out of the well. Similarly, the deeper the well of the Lennard-Jones potential is, the more stable the conformation between the two balls is. This is the key feature that we have used in inferring 3D chromosomal structures, which will be discussed later in detail.

The mathematical definition of the Lennard-Jones potential is:

(1) $$V_{LJ}=4\varepsilon \left[{\left(\frac{\sigma }{r}\right)}^{12}-{\left(\frac{\sigma }{r}\right)}^{6}\right]=\varepsilon \left[{\left(\frac{r_{m}}{r}\right)}^{12}-2{\left(\frac{r_{m}}{r}\right)}^{6}\right]$$

in which ε is the depth of the potential well, σ represents the distance at which the potential equals zero, r is the distance between the two beads, and $r_{m}$ is the distance at which the energy reaches the bottom of the energy well and *r_m_* = $2^{\frac{1}{6}}$σ ≈ 1.122σ. In other words, the value of the potential function is –ε when the distance is r_m_.

### 4.3. 2D Gaussian Function for Imputing Single-Cell Hi-C Contacts

The single-cell Hi-C contact matrix is sparse, which means that the number of 0s is much bigger than the number of s in the contact matrix. For example, there are ~400 1s in the single-cell Hi-C contact matrix for chromosome 1 of the mouse TH1 dataset while all other cells in the matrix are 0s. Each of the 1s indicates that there is at least one single-cell Hi-C contact observed between the bead pairs.

We used a 2D Gaussian function to impute the dominating zero values in the contact matrix as we previously did in [77], i.e., transform the 0s into non-zero values based on the distances to the location where a single-cell Hi-C contact is observed. The intuition behind this is that if beads *i* and *j* are in contact in the 3D space, the sequentially adjacent beads of these two beads should not be far away. For example, if beads *i* and *j* are proximate in the 3D space, the sequentially adjacent beads such as beads *i* − 1 and *i* + 1 should also be spatially close to, or at least not far away from, beads *j*, *j* + 1, and *j* − 1. The 2D Gaussian function we have used in this research is:

(2) $$\theta_{i,j}=\underset{\begin{array}{c} Hi-C\;\left(x_{p},y_{p}\right)=1\\ |x_{p}-i|\leq d_{0}\;and\;\left|y_{p}-j\right|\leq d_{0}\; \end{array}}{\sum }\exp\left(-\left(\frac{{\left(x_{p}-i\right)}^{2}}{\mu_{2}}+\frac{{\left(y_{p}-j\right)}^{2}}{\mu_{2}}\right)\right)$$

in which $x_{p},\;y_{p}$ indicate the location of a single-cell Hi-C contact in the contact matrix and parameter $\mu_{2}$ can be used to control the shape of the 2D Gaussian function. The larger the parameter $\mu_{2}$ is, the flatter the Gaussian-like curve would be, which means that the more bead pairs surrounding the single-cell Hi-C contact can be influenced. The $\left(x_{p}-i\right)$ and $\left(y_{p}-j\right)$ indicate the *x*-axis and *y*-axis distances from a zero value to the location that has a single-cell Hi-C contact. We use a parameter d_0_ to control how far the influence of a single-cell Hi-C contact can reach.

### 4.4. Loss Function

The following Equation (3) is the loss function that we newly designed based on the Lennard-Jones potential in Equation (1):

(3) $$E_{i,j}=\beta \times \theta_{i,j}{}^{n}\left[{\left(\frac{r_{m}}{d_{i,j}}\right)}^{12}-2\;{\left(\frac{r_{m}}{d_{i,j}}\right)}^{6}\right]$$

in which β is a scaling factor and set to 10, $\theta_{i,j}$ is the output of the 2D Gaussian function, $d_{i,j}$ is the distance between each bead pair in the 3D structure, and the value $r_{m}=2^{\frac{1}{6}}\sigma \approx 1.122\sigma$. We tested two different values for $r_{m}$. The first way is to set $r_{m}=8$, which means if two beads have their $\theta_{i,j}=1$, our algorithm will try to make their distance be 8 in the inferred 3D structure. Notice that SCL also uses 8 as the default value for the bead pairs having $\theta_{i,j}=1$. In this way, we can objectively compare the performance of our method and SCL. The second way is to set σ=8, which means $r_{m}=1.122\times 8=8.976$. If $d_{i,j}$ or the distance between any bead pairs is >16, we will not calculate the loss value using Equation (3) but directly assign a zero to the bead pair, that is, $E_{i,j}=0$. This is a similar idea to truncated Lennard-Jones potential, which can save computational time.

### 4.5. Initialization of Random 3D Chromosomal Structures

We used the same initialization step as in SCL [77]. Each chromosome is represented by a beads-on-a-string approach, that is, a chromosome is evenly divided into beads, and each DNA bead consists of a fixed number of DNA nucleotides or base pairs, e.g., 500 kb. A 3D lattice was created with the size of each side equaling 5*l* with *l* being the number of DNA beads for the whole chromosome. For example, the X-chromosome of the mouse contains 333 beads, making the size of the 3D lattice (5 × 333) × (5 × 333) × (5 × 333). Each small cubic in the 3D lattice has the size of 1 × 1 × 1. The 3D structure of a chromosome was initialized by randomly adding DNA beads one by one in a way that the newly added DNA bead must have a distance of [2,10] with $\sqrt{8}$ excluded [94] with all other currently existing beads in the 3D cubic lattice.

### 4.6. The Metropolis–Hastings Simulations

The cooling schedule defined in [95] was used in the simulated annealing process. The initial temperature was set as T_0_ = 10. The current temperature during each iteration of the simulated annealing process is $T_{c}=T_{0}\times {\left(0.9\right)}^{p}$ with *p* representing the number of times that the temperature has been decreased.

To stabilize the system at a certain temperature, a sufficient number of attempts were allowed at each temperature. If the number of attempts at a certain temperature reaches 100 times the number of beads or there are 10 accepted moves for each DNA bead on average [95], the temperature decreases based on the cooling schedule, followed by a new iteration of attempts. During each attempt, the Metropolis–Hastings algorithm was used to randomly select a DNA bead to randomly move from its current location to one of its 26 adjacent cells in the 3D cubic lattice (nine from the level above the current location, nine from the level below the current location, and eight from the same level of current location). The acceptance of move depends on ΔLoss, which is the change of the value of the loss function before and after the move: $\Delta Loss=Loss\left(after\right)-Loss\left(before\right)$. If ΔLoss ≤ 0, the move is accepted all the time. If ΔLoss > 0, the move is accepted with a probability P=e−ΔLossTc, where *T_c_* indicates the current temperature [95].

To generate high-resolution structures with 50 kb resolution, the 3D structure with 500 kb resolution was generated first. After that, nine beads were added in between every two consecutive 500 kb beads. These nine beads were added in the same way as in the initialization step. After that, we only applied 5*l* attempts with a constant temperature of 0.1, which is only to refine the structure without a large alternation.

### 4.7. Model Selection

A total of 50 structures were generated, and the one with the highest quality was selected and shown in the figures. We used TM-score to compare each of the models against all of the others and then calculated the average structural similarity. For each model, an average structural similarity is calculated. All models were ranked based on the average structural similarity, and the model ranked at No.1 or which had the highest average structural similarity was selected and used as the final output.

### 4.8. Computational Time

We implemented the system in C++, and the source code of the system can be found at http://dna.cs.miami.edu/LJ3D/. Benchmarked on an Intel Xeon CPU at 2.70 GHz, it took our Lennard-Jones method 7 min to model the 3D structure of the X-chromosome of a TH1 cell at 500 kb resolution, and 2 h 25 min for the structure at 50 kb resolution. In comparison, SCL needs 27 min and 3 h 17 min to model the structure of the same chromosome at 500 kb and 50 kb resolutions, respectively.

## 5. Conclusions

The Lennard-Jones potential can be used to infer 3D chromosomal structures based on single-cell Hi-C data. The well depth of the Lennard-Jones potential can be used to model how easy it is to break the distances between bead pairs. For the bead pairs that have a single-cell Hi-C contact observed, our loss function assigns a deeper well that makes it harder to be broken. For the bead pairs that do not have a single-cell Hi-C contact, we used a 2D Gaussian function to impute their contact values based on their distances to the other bead pairs that do have single-cell Hi-C contacts, and then the loss function assigns shallower well depths for those bead pairs. We benchmarked a set of different parameters from various perspectives and found the best parameter that results in the best performance.

## Data Availability

The software of LJ3D can be found at http://dna.cs.miami.edu/LJ3D/ (accessed on 30 May 2021).
